# Open Reduction in Traumatic Cervical Facet Dislocation Does Not Delay Time to Treatment

**DOI:** 10.7759/cureus.68955

**Published:** 2024-09-08

**Authors:** Noah M Yaffe, Collin M Labak, Pranav Kumar, Eric Herring, Dustin J Donnelly, Gabriel Smith

**Affiliations:** 1 Neurological Surgery, University Hospitals Cleveland Medical Center, Cleveland, USA

**Keywords:** cervical facet dislocation, neurosurgery, spine surgery, spine trauma, trauma

## Abstract

Background

Cervical facet dislocation is a serious injury that can result in permanent neurologic damage. Current guidelines recommend immediate closed reduction of cervical dislocations, though the efficacy of this practice remains a debate. This study aims to evaluate whether immediate open reduction and fixation of cervical dislocations offer equal or better outcomes for patients and limit the need for follow-up operations.

Methods

This is a retrospective study including patients who presented to the emergency department of a single institution from 2008 to 2023 with cervical facet dislocation. Patients were divided into groups based on initial treatment: either open or closed reduction. Time to surgery was calculated as the time between arrival to the ED and incision time in the OR. Primary outcomes were improvement in motor and sensory deficits at six-week post-operative follow-up.

Results

There were 31 patients who met the inclusion criteria. Time to treatment did not differ significantly between the open versus closed reduction groups. There were no differences between groups in improvement in motor function, sensory function, or pain at the six-week follow-up. All patients treated with initial closed reduction ultimately required surgical stabilization.

Conclusions

Open reduction as a first-line treatment did not increase the time to treatment for patients with cervical facet dislocations. Patients had equivalent functional outcomes in both treatment groups. The findings suggest that current practice guidelines may delay definitive treatment without improving patient safety or outcomes.

## Introduction

Traumatic cervical spine facet dislocation is a serious injury that can result in permanent neurologic damage [1]. The mechanism of this injury is due to distraction and flexion force vectors on the spine with bony injury related to rotational force [2-4]. Depending on the level and severity of injury, neurologic complications range from mild radiculopathy to complete quadriplegia with ventilator dependence [1]. Patients with signs of neurologic compromise require urgent surgical attention and treatment typically involves closed or open reduction.

Current joint guidelines from the Congress of Neurological Surgeons (CNS) and the American Association of Neurological Surgeons (AANS) recommend prompt closed reduction of cervical dislocations in patients who are awake and alert, though this remains a debate in the literature [5]. This class of pathology generally requires follow-up stabilization surgery regardless of neurologic status given inherent concerns of cervical spinal instability [6,7]. In addition, the initial closed reduction may put patients at risk of transient or permanent neurologic deficits, including complete spinal cord injury, though these complications are rare [5,8-12]. Therefore, there is a need for further evaluation of the acute management of cervical facet dislocations.

This study aimed to determine if the time needed to achieve open reduction was comparable to the time needed to achieve closed reduction at an academic hospital with a Level 1 trauma center. We also aimed to compare neurologic outcomes in patients who underwent closed reduction compared to those who underwent open reduction as a first treatment option. We finally sought to describe the frequency with which patients who received closed reduction underwent subsequent operative management.

## Materials and methods

**Figure 1:**
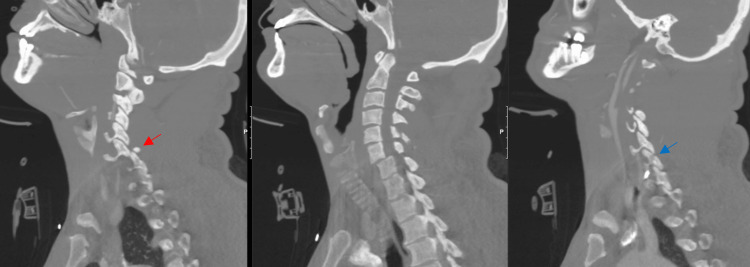
CT of the cervical spine with right parasagittal, midsagittal, and left parasagittal views of a cervical fracture/dislocation with a right C6-7 perched facet (left image, red arrow) and left C6-7 lateral mass fracture (right image, blue arrow).

**Figure 2:**
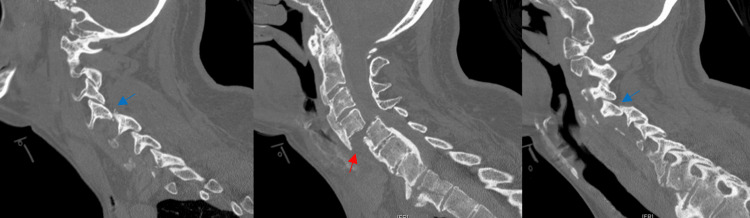
CT of the cervical spine on a patient with diffuse idiopathic skeletal hyperostosis from the data set demonstrating fracture/dislocation at the C4-5 level (center, red arrow), and bilateral perched facet joints (left, right panes, blue arrows).

**Figure 3:**
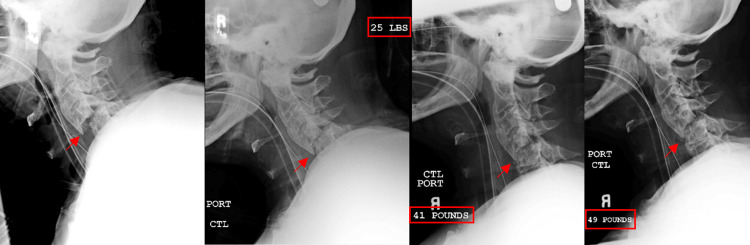
Sequential lateral plain film radiographs showing the patient undergoing closed reduction with sequential increase in applied weight. The amount of weight is denoted in each film with a red box. A fracture line is demonstrated in each pane with a red arrow.

**Figure 4:**
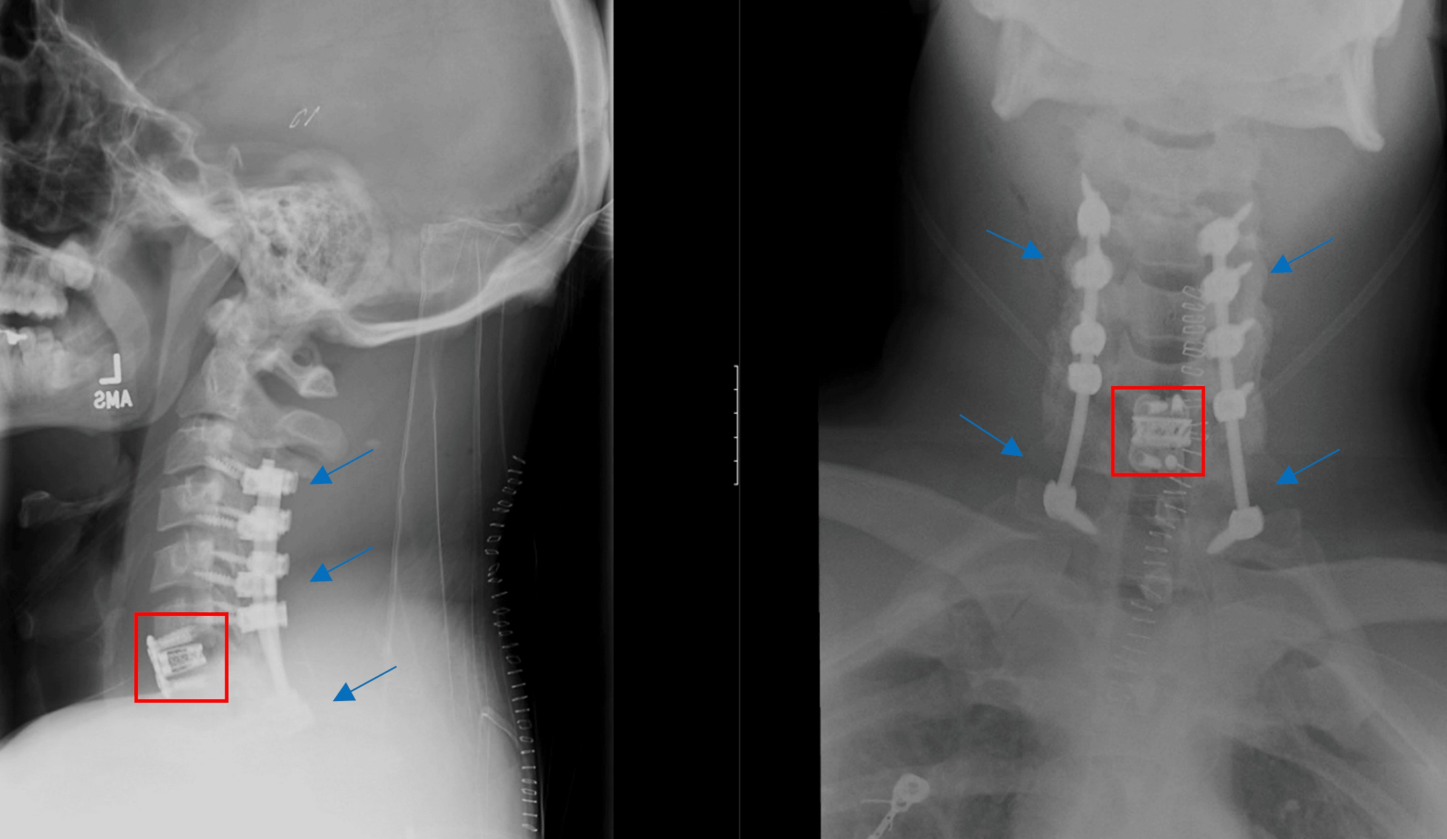
Lateral (left) and anterior-posterior (right) plain films of surgical construct, showing C6-7 anterior cervical discectomy and fusion (red box) with C3-T1 posterior cervical decompression and fusion (blue arrows).

This retrospective cohort study was performed at a single tertiary care institution with a Level 1 trauma center. Records were obtained from 2008 to 2023 with the aid of an institutional Trauma Registry. Included patients met the following criteria: diagnosis of cervical facet dislocation with or without fracture, treatment with a closed or open reduction during index hospitalization, and presentation to the emergency department of the health system’s main campus with Level 1 trauma center. Patients were excluded if there was evidence of injury at the level of C2 or higher if they presented with altered mental status, or if patients had additional rostral injuries as a separate set of guidelines governs these cases. Patients under the age of 18 were also excluded. Radiographic images of example injuries are displayed in Figures 1, 2. Radiographic images of patients after closed and open reduction are included in Figures 3, 4.

Collected variables included date and time of presentation, initial treatment (i.e., closed versus open reduction), date and time of initial reduction, injury details, presenting symptoms including American Spinal Injury Association (ASIA) Impairment Scale, presence of neurologic recovery and ASIA Scale at six-week follow-up, complications, comorbidities, and demographics. Patients were divided into a closed reduction group or an open reduction group based on the initial treatment they received. Additional subgroup analysis categorized patients into unilateral versus bilateral injury groups. Data were collected and stored using REDCap electronic data capture tools (Vanderbilt University, Nashville, TN) hosted at University Hospitals (grant: UL1TR002548) [13,14]. Descriptive statistics were performed to characterize the dataset. Independent t-tests were used to compare time-to-treatment between groups. Chi-square tests were used to evaluate patient outcomes between groups. Statistical analysis was conducted with Excel (version 16.80; Microsoft, Redmond, Washington, DC) and JMP (version 17.0.0; JMP Statistical Discovery LLC, Cary, NC). All data were collected through the electronic medical record under University Hospitals' IRB approval study number 20231238.

## Results

There were 31 patients who met inclusion criteria: 27 in the open reduction group and four in the closed reduction group. The mean age of participants was 45 years, and the cohort was predominantly male (74%). There were no statistically significant differences in demographic factors between the two study groups. Full demographic information is reported in Table 1.

**Table 1:** Cohort characteristics T2DM=Type 2 Diabetes Mellitus; CAD=Coronary Artery Disease; COPD=Chronic Obstructive Pulmonary Disease; CKD=Chronic Kidney Disease; DISH=Diffuse Idiopathic Skeletal Hyperostosis; AS=Ankylosing Spondylitis

	Open reduction (n=27)	Closed reduction (n=4)	Total (n=31)
Mean age	42.4	64.8	45.3
Male sex	20 (74%)	3 (75%)	23 (74%)
Race and ethnicity			
Hispanic	1 (4%)	-	1 (3%)
White	16 (59%)	3 (75%)	19 (61%)
Black	10 (37%)	1 (25%)	11 (35%)
Comorbidities			
Hypertension	11 (41%)	1 (25%)	12 (39%)
T2DM	1 (4%)	1 (25%)	2 (6%)
CAD	1 (4%)	1 (25%)	2 (6%)
Asthma or COPD	2 (7%)	-	2 (6%)
DISH/AS	1 (4%)	1 (25%)	2 (6%)

Eighty-one percent of patients had concomitant fracture and dislocation. High-impact and low-impact injuries occurred roughly evenly and there was one case of non-traumatic dislocation. The most common level of injury was C6. Patients in this cohort were more likely to have bilateral facet dislocations, though this was not statistically significant. All patients received computed tomography (CT) scans of the cervical spine. Seventy-four percent of patients had magnetic resonance imaging (MRI) of the cervical spine prior to treatment. Injury details are listed in Table 2. There were no significant differences between the treatment groups in any of these categories.

**Table 2:** Injury details CT=Computed Tomography; MRI=Magnetic Resonance Imaging

	Open reduction (n=27)	Closed reduction (n=4)	Total (n=31)
Injury type			
Dislocation	5 (19%)	1 (25%)	6 (19%)
Dislocation + fracture	22 (81%)	3 (75%)	25 (81%)
Mechanism			
High impact	12 (44%)	2 (50%)	14 (45%)
Low impact	14 (52%)	2 (50%)	16 (52%)
Atraumatic	1 (3%)	-	1 (3%)
Highest spinal level			
C3	4 (15%)	-	4 (13%)
C4	4 (15%)	1 (25%)	5 (16%)
C5	8 (30%)	1 (25%)	9 (29%)
C6	10 (37%)	2 (50%)	12 (39%)
C7	1 (4%)	-	1 (3%)
Laterality			
Unilateral	12 (44%)	1 (25%)	13 (42%)
Bilateral	15 (56%)	3 (75%)	18 (58%)
Imaging workup			
CT	27 (100%)	4 (100%)	31 (100%)
MRI	21 (78%)	2 (50%)	23 (74%)

Time to treatment

The mean time-to-treatment with open reduction was 9.2 +/- 5.4 hours. The mean time-to-treatment with closed reduction was 9.9 +/- 5.8 hours. There was no significant difference in time-to-treatment between these groups (t(3.8) = -0.23, p = 0.83). Patients with evidence of more severe spinal cord injuries - ASIA grades A, B, or C - had a mean time-to-treatment with open reduction of 6.1 +/- 2.8 hours.

Functional outcomes

Overall, there was no significant difference in symptom improvement between treatment groups. Improvement in sensory symptoms did not differ significantly, X2 (1, N = 31) = 2.18, p = 0.14. There was no difference in the improvement of motor weakness, X2 (1, N = 31) = 1.13, p = 0.29. All patients who presented with pain had improvement at six-week follow-up regardless of treatment modality. Patients with concomitant spinal cord injury most commonly presented with ASIA grade D or E injuries. All patients had equal or improved ASIA grades at follow-up. Every patient who was initially treated with closed reduction went on to receive definitive surgical fixation at a later date. Of note, all of these corrective surgeries occurred before the six-week follow-up appointment after the initial injury. Outcomes data are reported in Table 3.

**Table 3:** Outcomes data *The percentages for each group exceed 100% as patients presented with multiple symptoms. **The total number of patients with ASIA grades at presentation and follow-up were different from the total number of patients in the open and closed reduction groups presented in other areas of this table due to lack of cord injury.

	Open reduction	Closed reduction	Total
Presenting symptoms			
Pain	22 (81%)	4 (100%)	26 (84%)
Motor weakness	15 (56%)	3 (75%)	18 (58%)
Sensory loss	14 (52%)	2 (50%)	16 (52%)
% with symptom resolution at follow-up^*^			
Pain	100%	100%	100%
Motor weakness	91%	67%	86%
Sensory loss	91%	50%	85%
ASIA grade at presentation^**^			
A	3	1	4
B	0	0	0
C	1	0	1
D	11	0	11
E	6	2	8
ASIA grade at follow-up^**^			
A	2	0	2
B	0	0	0
C	1	0	1
D	5	0	5
E	12	3	15
Completion of rehab program			
Yes	6 (22%)	1 (25%)	7 (23%)
No	21 (78%)	3 (75%)	24 (77%)

Procedural complications

There were three complications in this study. In the open reduction group, one patient had a recurrent dislocation, and one patient had a postoperative infection. There was one major complication in the closed reduction group. A patient presenting without motor symptoms experienced an immediate loss of bilateral lower extremity motor and sensory function when closed reduction was attempted. This had resolved at follow-up after surgical open reduction and stabilization.

Unilateral vs. bilateral injury subgroup analysis

The mean time-to-treatment with open reduction for unilateral injuries was 9.9 +/- 5.4 hours. The mean time-to-treatment with open reduction for bilateral injuries was 8.5 +/- 5.6 hours. This difference was not statistically significant (t(24) = -0.66, p = 0.51).

Improvement in sensory symptoms did not differ significantly between the unilateral and bilateral injury groups, X^2^ (1, N = 13) = 0.13, p = 0.72. There was also no difference in improvement of motor weakness, X2 (1, N = 14) = 0.05, p = 0.83. All patients experienced improvement in pain regardless of injury laterality. ASIA grade on presentation did not differ significantly between the injury groups, X^2^ (3, N = 24) = 3.84, p = 0.28. Surgical approach - anterior, posterior, or combined - did not differ significantly between the unilateral and bilateral injury groups, X^2^ (2, N = 27) = 1.16, p = 0.56.

## Discussion

The findings of this study demonstrate that there was no statistically or clinically significant difference in time-to-treatment between patients who received initial closed reduction and patients who received initial open reduction. The initial treatment did not impact the resolution of patients’ neurologic symptoms at a six-week follow-up. However, it is worth noting that every patient in the closed reduction group received definitive surgical intervention at a later date, and all of these surgeries occurred before the six-week follow-up timepoint. This makes it difficult to conclude whether the closed reduction had a positive impact on symptoms or if improvements occurred in response to surgery. All patients in the open reduction group had an equal or improved ASIA grade at follow-up. This was not significantly different from the closed reduction group. In total, open reduction led to comparable outcomes across multiple domains without delaying time to stabilization after acute injury.

The overall positive outcomes of the open reduction cohort highlight the feasibility of open reduction as a first-line treatment for cervical facet dislocations. For institutions with the capability to operate on these patients in the acute setting, initial open reduction accelerates a patient’s time to definitive treatment. Immediate fixation also avoids any harm that may occur due to unresolved ligamentous injury in patients who undergo initial closed reduction alone. These beneficial outcomes suggest a need to further evaluate open reduction as an initial treatment for facet dislocations to better inform existing guidelines.

Though the literature on this subject is sparse, these results are consistent with existing findings. A systematic review investigating unilateral facet dislocations found that non-operative management had an 80% treatment failure rate compared to a 2.6% failure rate in operative management [15]. Patients managed non-operatively often required surgery at a later date to improve neurologic symptoms and pain, which is consistent with other reports [16]. Notably, the majority of studies included in this review are 15-30 years old and there is little in the way of new evidence on this question, though similar studies support the safety of open reduction as an initial treatment strategy [17,18]. Due to this paucity of relevant data, existing guidelines only comment on the validity of closed reduction rather than comparing closed to open techniques directly [5]. All of this points to a clear gap in the literature regarding the efficacy of immediate open reduction for cervical spine facet dislocation.

The comparable time-to-treatment in both groups of approximately nine to 10 hours conflicts with previously reported data. Prior studies comparing outcomes between open and closed reduction have reported varied time frames, with one article finding a median time to open reduction of 22 hours compared to six hours for closed reduction [19]. Other studies suggest optimal timing for decompression to be within four hours of injury, with more conservative estimates still arguing that the best outcomes may be achieved if treatment occurs within eight hours [20,21]. These findings would suggest that closed reduction may be the only reasonable choice for urgent decompression, however, our data show that open reduction is feasible within a similar amount of time, even meeting a strict <8-hour cutoff for the most severe injuries. Given the disparities in treatment times found in this study compared to prior literature, further multi-institutional work is required to better characterize realistic timelines.

It is also likely that institutional differences in these studies have resulted in an overestimation of time-to-surgery and an underestimation of time-to-closed reduction. This would skew reported neurologic outcomes. For patients at a Level 1 trauma center, like in our study, open reduction may be the superior choice when considering all factors. This is not to say that closed reduction has no place in care, as it may remain the sole option for patients presenting to a regional center prior to transfer to a higher level of care, or in cases where the patient may not be suitable for operative intervention at that time. Existing studies have highlighted the importance of maintaining this skill set for physicians practicing in those centers [22].

Another institutional consideration for differences in time-to-treatment is the high elective case volume at our hospital that necessitates a later start time for add-on cases that are not declared emergencies. Given that many of the patients in our study were ASIA grade E on presentation, they required fracture reduction and stabilization as opposed to decompression of bony elements off of the spinal cord. The consulting surgeon may have felt it appropriate to add on these open reductions after the surgical day, or to schedule them as the first case for the morning if the patient came in overnight. In these situations, there is a reasonable benefit to waiting if it means the surgeon can work with their standard operating room team. This may explain the variance in time-to-open reduction observed in this study.

Subgroup analysis comparing unilateral and bilateral injuries found no significant differences in time-to-treatment with open reduction, surgical approach, ASIA grade on presentation, or functional outcomes. These results may seem unexpected. Bilateral injuries typically involve more substantial damage to posterior osteo-ligamentous structures when compared to unilateral facet dislocations and infer a higher risk of cord injury [23]. For this reason, there is concern that anterior cervical discectomy and fusion (ACDF) alone is inadequate for proper stabilization [24,25], though this is a nuanced decision as a pure posterior approach risks cord injury from a herniated disc that is often comorbid with these injuries. The absence of significant differences in treatment between our patients is likely due to two main factors: 1) our patients presented with similar ASIA grades, suggesting that the bilateral group may have had intact posterior structures and did not require a different surgical approach, and 2) the sample size of the study limits statistical power. Future studies of larger cohorts remain warranted.

The primary limitation of this study is the small number of closed reduction cases, which has a sample size of four. The goal of our original methodology was to obtain comparably sized cohorts, but a review of the medical record identified many cases of cervical spine dislocation with concomitant cranial injury that made patients at our institution ineligible for inclusion in this study. Though this limits our ability to perform some statistical analyses, the primary conclusions are still valid. The data shows a 0.72-hour (43 minutes) difference in time-to-treatment between groups. A power analysis - assuming a statistical power of only 0.80 - reveals that one would need over 1,700 patients to measure statistical significance at that effect size, which is a far higher number of patients than one would expect to encounter with this type of injury in a 15-year period at a single institution. In addition, the time difference between groups is ~7%-8% of time-to-treatment, so it is uncertain whether this difference could be considered clinically significant regardless of the statistical outcome. With this in mind, it is valid for the primary conclusion of this study to be that there was no substantial difference in time-to-treatment between the open and closed reduction groups. Given limitations on sample size, we have chosen not to draw statistical conclusions about complication rates between treatment options despite marked differences.

A qualitative analysis of complications in this study is still warranted. One patient in the closed reduction group deteriorated from ASIA grade E to grade A when reduction was attempted. A similar case was reported in another study of traumatic cervical spine facet dislocation [26]. Upon initiation of traction, the patient had complete loss of bilateral lower extremity motor and sensory function and became hypotensive, requiring immediate surgical stabilization. The possibility of such severe complications warrants reconsideration of the most appropriate treatment for this injury. While neurological deterioration from closed reduction may be rare, it is unnecessary to expose patients to this risk when open reduction is available in a similar timeframe, when it may reduce the risk of deterioration, and when surgical fixation is a likely outcome for most patients undergoing closed reduction regardless.

## Conclusions

Current guidelines recommend immediate closed reduction for traumatic cervical spine facet dislocations in patients who are awake and alert, though most of these patients go on to require surgical fixation for these injuries. This single-institution study found no statistically or clinically significant differences in time-to-treatment between open and closed reduction for these injuries, demonstrating that initial open reduction avoids delays in definitive management and eliminates the risk of rare but serious complications of closed reduction. Further multi-institutional studies are recommended to better inform neurosurgical practice patterns and guidelines.
